# 

**Published:** 1879-09

**Authors:** 


*CONTENTS.*

| | Page |
|---|---|
| Going up Stairs | 1 |
| Value of Vaccination | 2 |
| A Gentleman | 3 |
| Jew and Gentile | 4 |
| Work and Worry | 5 |
| The Uses of the Tongue | 6 |
| Cleanliness | 7 |
| Fever and Ague Destroyer | 7 |
| Eye Diseases | 9 |
| Worth of a Wag | 10 |
| Condiments | 12 |
| Sick Rooms | 14 |
| Drunkards | 15 |
| Quinine and Castor Oil | 16 |
| Longevity and Physicians | 16 |
| Boils and Whitlows | 17 |
| Fidelity | 17 |
| Man's Rights | 18 |
| Household Knowledge | 19 |
| Useful Items | 21 |
| Weights and Measures | 21 |
| Husband—Wife, Spinster, Lady | 22 |
| The Heirloom | 22 |
| How to Live | 23 |
| For the Farm and Family | 24 |
| Teaching Music | 26 |
| The Two Newsboys | 26 |
| Wall Paper Poison | 27 |
| Preservative | 27 |
| The Two Revenges | 28 |
| Making Coal Fires | 29 |
| Origin of Plants | 30 |
| The Monster Monopoly | 30 |

